# Equations to Predict Growth Performance Changes by Dietary Deoxynivalenol in Pigs

**DOI:** 10.3390/toxins13050360

**Published:** 2021-05-19

**Authors:** Jongkeon Kim, Jin Young Jeong, Jung Yeol Sung, Beob Gyun Kim

**Affiliations:** 1 Department of Animal Science and Technology, Konkuk University, Seoul 05029, Korea; gun5233@konkuk.ac.kr; 2 Animal Nutritional Physiology Team, National Institute of Animal Science, Rural Development Administration, Wanju-gun, Jeonju-si 55363, Jeollabuk-do, Korea; jeong73@korea.kr; 3 Department of Animal Sciences, Purdue University, West Lafayette, IN 47907, USA; sung73@purdue.edu

**Keywords:** deoxynivalenol, equations, growth performance, pigs

## Abstract

The objectives of the present work were to assess the accuracy of previously published equations for predicting effects of deoxynivalenol (DON) on the growth performance changes of pigs and to update equations based on recently published data. A total of 59 data were employed for the validation of previously published equations. These data were used to update the equations. The REG and CORR procedures of SAS were used. In the present validation test, a linear bias was significant (*p* < 0.05), indicating that prediction errors were not consistent across the data ranges. The intercept for ΔFI (−7.75 ± 1.19, *p* < 0.01) representing a mean bias was less than 0, indicating that the predicted mean of ΔFI was greater than the measured mean of ΔFI. Dietary DON concentrations had negative correlations with ΔWG (*r* = −0.79; *p* < 0.01) and ΔFI (*r* = −0.71; *p* < 0.01). Updated prediction equations were: ΔWG = −5.93 × DON with *r*^2^ = 0.77 and ΔFI = −4.42 × DON with *r*^2^ = 0.68. In conclusion, the novel equations developed in this study might accurately predict effects of dietary DON on the performance changes of pigs.

## 1. Introduction

Frequent occurrence of mycotoxins in feedstuffs has been reported due to fungal growth before harvesting of grains and their long-term storage in a humid climate [1,2]. Deoxynivalenol (DON), one of mycotoxins generated by the *Fusarium* fungus, can decrease feed intake (FI) that cause growth retardation in weaning pigs [3]. Moreover, DON can decrease the ileal digestibility of lysine, threonine, and tryptophan in corn-soybean meal-based diets [4] and villus height of jejunum in pigs [5,6]. In addition to these detrimental effects, dietary DON can impair the cell proliferation, immune system, and normal function of organs in pigs [7]. Consequently, the occurrence of DON in feed negatively affects growth performance, nutrient utilization, and physiological status of pigs.

In pig production, growth performance is important for economic profits of commercial farms. To predict the influence of DON on growth performance of pigs, Mok et al. [8] reported equations for the changes of FI and weight gain (WG) of pigs depending on dietary DON concentrations. Since then, quite a few data for the effects of dietary DON on the performance of pigs have been reported. However, these equations have not been validated for the accuracy. Therefore, the objectives of this study were to evaluate the accuracy of previously published equations for predicting effects of DON on the growth performance of pigs and to update the equations with recent data.

## 2. Results

### 2.1. Validation of Previously Published Equations

**Figure 1:**
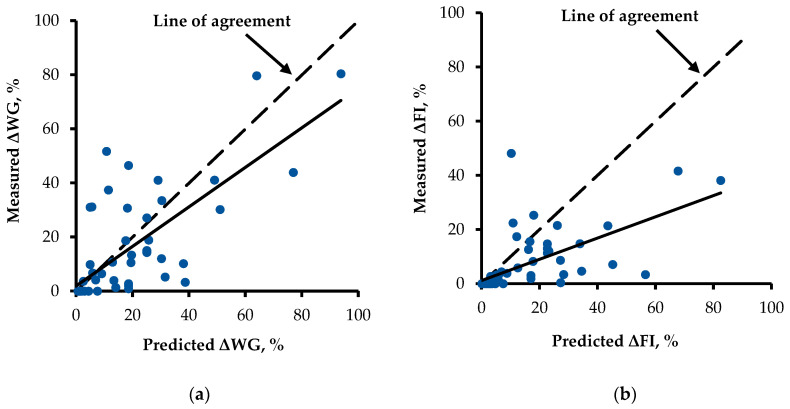
Validation of equations suggested by Mok et al. [8] for weight gain changes (ΔWG, %) and feed intake changes (ΔFI, %) by dietary deoxynivalenol concentration in feeds (mg/kg). A total of 59 data from 22 experiments published between 2013 and 2020 were used. Based on regression analyses of measured minus predicted ΔWG (%) and ΔFI (%) on the predicted ΔWG (%) and ΔFI (%), respectively, adjusted to the mean as 0. (**a**) The slope (−0.27; SE = 0.09; *p* < 0.01) was less than 0 and the intercept (−1.94; SE = 1.77; *p* = 0.276) was not different from 0 for ΔWG (%). (**b**) In addition, the slope (−0.61; SE = 0.07; *p* < 0.01) was less than 0 and the intercept (−7.75; SE = 1.19; *p* < 0.01) was less than 0 for ΔFI (%).

Based on the validation test, a linear bias was significant (*p* < 0.05) in the equations suggested by Mok et al. [8], indicating that the difference between the predicted values and the measured values were not consistent across the data ranges (Figure 1). The intercept for ΔFI (−7.75 ± 1.19, *p* < 0.01) representing a mean bias was less than 0, indicating that the predicted mean value of ΔFI was greater than the measured mean value of ΔFI.

### 2.2. Determination of Correlation Coefficients among Variables

Dietary DON concentrations were negatively correlated with ΔWG (*r* = −0.79, *p* < 0.01; Table 1) and ΔFI (*r =* −0.71, *p* < 0.01).

### 2.3. Determination of the Equations for Predicting Growth Performance of Pigs

**Figure 2:**
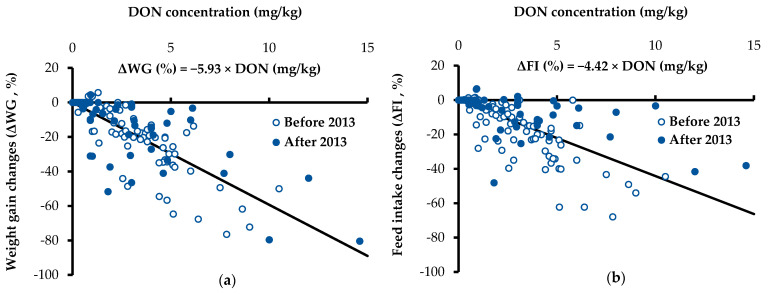
Regression equations for predicting weight gain changes (ΔWG, %) and feed intake changes (ΔFI, %) of pigs by dietary deoxynivalenol (*n* = 156). The y-intercept of new equations was forced to zero. (**a**) ΔWG = −5.93 × DON with SE of the slope = 0.26, *r*^2^ = 0.77, and *p* < 0.001. (**b**) ΔFI = −4.42 × DON with SE of the slope = 0.25, *r*^2^ = 0.68, and *p* < 0.001. Dietary DON concentrations ranged from 0 to 14.6 mg/kg.

Prediction equations for estimating ΔWG and ΔFI were: ΔWG = −5.93 × DON with *r*^2^ = 0.77 and ΔFI = −4.42 × DON with *r*^2^ = 0.68, respectively. Dietary DON concentrations ranged from 0 to 14.6 mg/kg (Figure 2).

## 3. Discussion

Deoxynivalenol is called vomitoxin as this toxin potentially induces vomiting in pigs particularly at high concentrations [9]. Dietary DON can decrease the WG of pigs mainly due to reduced FI [8,10,11,12]. Quantitative estimation of WG and FI changes by dietary DON is important in the swine production industry. Prediction equations for estimating ΔWG and ΔFI by dietary DON are available in the literature [8,13]. Based on the present validation study employing recent 8 years of data, old equations [8,13] were not very accurate. The validation of these equations for ΔWG and ΔFI reported by Mok et al. [8] showed that the slope representing a linear bias was less than 0 in both equations, indicating that predicted ΔWG and ΔFI values increasingly deviated from measured ΔWG and ΔFI values on the basis of predicted mean values across the range of data. The reason for such a linear bias in these prediction equations is unclear. Stronger effects of natural DON in feeds compared with purified DON might have resulted in the bias in the validation. Natural occurrences of DON in feed ingredients are often accompanied by other mycotoxins such as zearalenone [2,14]. The co-occurrence of DON and zearalenone in diets potentially further decease the WG in pigs compared with only DON contamination in diets fed to pigs [15,16]. However, the equation models for predicting the effects of co-occurrence of DON and zearalenone on growth performance in pigs were not developed due to the limited information of ZEN concentrations in the collected data from the literature. The intercept representing a mean bias was less than 0 in the prediction equation for ΔFI based on the validation of the equations by Mok et al. [8], indicating that the ΔFI values of pigs fed dietary DON were overestimated by the prediction equation.

The negative correlation between dietary DON and ΔFI in the present work (Table 1) is in agreement with previous reports [3,6]. The steepness of the slope in these prediction equations (Figure 2) was less than the slope reported by Mok et al. [8], indicating that these novel equations developed with additional recent data would estimate less ΔWG and ΔFI per DON concentration compared with previously published prediction equations [8]. Theoretically, no change of WG or FI is expected if a diet contains no DON. Previous equations reported by Mok et al. [8] and Andretta et al. [13] had no or negligible intercepts. In these novel equations, thus, intercepts were forced to zero. Consequently, based on the large number of observations including the recent data, these updated equations might be able to predict the growth performance better than the previous ones in a wide range of dietary DON concentrations.

## 4. Conclusions

In conclusion, the novel equations developed in this study based on a large number of observations including recent data might accurately predict effects of dietary deoxynivalenol on the weight gain and feed intake changes of pigs.

## 5. Materials and Methods

### 5.1. Data Collection

Google Scholar and Scopus were used for literature search. Keywords used were growth performance, deoxynivalenol, and pigs. The studies found through the literature search were then manually screened based on the title and experimental information. The papers with DON concentrations in the diet, FI, WG, initial BW, final BW, sex, number of pigs, and experimental period were selected for the database. The WG or FI of pigs was calculated based on the feed efficiency when there was limited information on WG or FI in the literature.

A total of 59 data from 22 studies published between 2013 and 2020 reporting the effects of dietary DON on the growth performance changes of pigs were used to validate the previously published equations [8]. In addition to the data (97 observations) published before 2013, recently published data (Supplementary Materials List S1) were pooled to develop novel equations for estimating ΔWG and ΔFI by dietary DON in pigs. The prediction equations based on the DON analysis method were provided in Supplementary Figures S1–S4.

### 5.2. Calculation and Statistical Analysis

The changes (%) of WG or FI relative to the control diet group were calculated as follows:

(1) $$\Delta \mathrm{WG}\;\mathrm{or}\;\Delta \mathrm{FI}\;(\%)=\frac{\mathrm{WG}\;\mathrm{or}\;\mathrm{FI}\;\mathrm{of}\;\mathrm{DON}\;\mathrm{treatment}\;\mathrm{group}\;-\;\mathrm{WG}\;\mathrm{or}\;\mathrm{FI}\;\mathrm{of}\;\mathrm{control}\;\mathrm{group}}{\mathrm{WG}\;\mathrm{or}\;\mathrm{FI}\;\mathrm{of}\;\mathrm{control}\;\mathrm{group}}\;\times \;100$$

The calculated ΔWG and ΔFI data were shifted symmetrically on the basis of the origin. The accuracy of the previously published equations [8] was assessed by regressing the measured ΔWG and ΔFI values obtained in the literature minus the predicted ΔWG and ΔFI values on each predicted value centered to the mean [17,18] using the REG procedure of SAS (SAS Inst. Inc., Cary, NC, USA). In this linear regression, the intercept and the slope represented a mean bias and a linear bias, respectively.

The REG procedure of SAS was used to develop novel linear equations after confirming no quadratic relationship between DON concentrations and ΔWG or ΔFI values. The y-intercept of new equations was forced to zero using NOINT option in SAS. All data were analyzed using the CORR procedure of SAS to determine correlation coefficients among variables. The statistical significance was declared at an alpha less than 0.05.

## Figures and Tables

**Table 1:** Correlations between dietary deoxynivalenol (DON) concentrations, weight gain changes (ΔWG), feed intake changes (ΔFI), initial body weight (BW), mean BW, and experimental period.

Item	ΔWG	ΔFI	Initial BW	Mean BW	Experimental Period, Day
DON	−0.79 **	−0.71 **	−0.01	−0.05	−0.04
ΔWG		0.85 **	−0.03	0.06	0.17
ΔFI			−0.03	0.01	0.05
Initial BW				0.94 **	0.22 **
Mean BW					0.52 **

** *p* < 0.01.

## Data Availability

The data presented in this work are available.

## Supplementary Materials

The following are available online at https://www.mdpi.com/article/10.3390/toxins13050360/s1, List S1: List of cited references to update the prediction equations; Table S1: Experimental conditions in 42 studies. Figure S1: Regression equations for predicting weight gain changes (ΔWG, %) and feed intake changes (ΔFI, %) of pigs by dietary deoxynivalenol based on ELISA analysis method (*n* = 24); Figure S2: Regression equations for predicting weight gain changes (ΔWG, %) and feed intake changes (ΔFI, %) of pigs by dietary deoxynivalenol based on HPLC analysis method (*n* = 57). Figure S3: Regression equations for predicting weight gain changes (ΔWG, %) and feed intake changes (ΔFI, %) of pigs by dietary deoxynivalenol based on LC–MS/MS analysis method (*n* = 62); Figure S4: Regression equations for predicting weight gain changes (ΔWG, %) and feed intake changes (ΔFI, %) of pigs by dietary deoxynivalenol based on unknown analysis method (*n* = 13).
