# Supplementary material for: Equations to Predict Growth Performance Changes by Dietary Deoxynivalenol in Pigs
Source: Toxins (Basel). 2021 May 19;13(5):360. doi: 10.3390/toxins13050360 (PMC8158744; doi:10.3390/toxins13050360)
Supplement: Supplementary file 1 [file toxins-13-00360-s001.zip › toxins-1229250-supplementary header.pdf]

Supplementary Materials: Equations to Predict Growth Performance Changes by Dietary Deoxynivalenol in Pigs

Jongkeon Kim, Jin Young Jeong, Jung Yeol Sung and Beob Gyun Kim

List S1: List of cited references to update the prediction equations**.** A total of 59 data from 22 studies published between 2013 and 2020 reporting the effects of dietary deoxynivalenol on the growth performance changes of pigs were used to validate the previously published equations.

1. Friend, D.W.; Trenholm, H.L.; Fiser, P.S.; Hartin, K.E.; Thompson, B.K. Effect on dam performance and fetal development of deoxynivalenol (vomitoxin) contaminated wheat in the diet of pregnant gilts. *Can. J. Anim. Sci.* **1983**, *63*, 689–698.
2. Young, L.G.; Mcgirr, L.; Valli, V.E.; Lumsden, J.H.; Lun, A. Vomitoxin in corn fed to young pigs. *J. Anim. Sci.* **1983**, *57*, 655–664.
3. Chavez, E.R. Vomitoxin-contaminated wheat in pig diets: Pregnant and lactating gilts and weaners. *Can. J. Anim. Sci.* **1984**, *64*, 717–723.
4. Cote, L.M.; Beasley, V.R.; Bratich, P.M.; Swanson, S.P.; Shivaprasad, H.L.; Buck, W.B. Sex-related reduced weight gains in growing swine fed diets containing deoxynivalenol. *J. Anim. Sci.* **1985**, *61*, 942–950.
5. Lun, A.K.; Young, L.G.; Lumsden, J.H. The effects of vomitoxin and feed intake on the performance and blood characteristics of young pigs. *J. Anim. Sci*. **1985**, *61*, 1178–1185.
6. Pollmann, D.S.; Koch, B.A.; Seitz, L.M.; Mohr, H.E.; Kennedy, G.A. Deoxynivalenol-contaminated wheat in swine diets. *J. Anim. Sci.* **1985**, *60*, 239–247.
7. Foster, B.C.; Trenholm, H.L.; Friend, D.W.; Hartin, K.E.; Thompson, B.K. Evaluation of different sources of deoxynivalenol (vomitoxin) fed to swine. *Can. J. Anim. Sci.* **1986**, *66*, 1149–1154.
8. Bergsjø, B.; Langseth, W.; Nafstad, I.; Jansen, J.H.; Larsen, H.J.S. The effects of naturally deoxynivalenol-contaminated oats on the clinical condition, blood parameters, performance and carcass composition of growing pigs. *Vet. Res. Commun.* **1993**, *17*, 283–294.
9. He, P.; Young, L.G.; Forsberg, C. Microbially detoxified vomitoxin-contaminated corn for young pigs. *J. Anim. Sci.* **1993**, *71*, 963–967.
10. Rotter, B.A.; Thompson, B.K.; Lessard, M. Effects of deoxynivalenol-contaminated diet on performance and blood parameters in growing swine. *Can. J. Anim. Sci.* **1995**, *75*, 297–302.
11. Smith, T.K.; McMillan, E.G.; Castillo, J.B. Effect of feeding blends of fusarium mycotoxin-contaminated grains containing deoxynivalenol and fusaric acid on growth and feed consumption of immature swine. *J. Anim. Sci.* **1997**, *75*, 2184–2191.
12. Swamy, H.V.L.N.; Smith, T.K.; MacDonald, E.J.; Boermans, H.J.; Squires, E.J. Effects of feeding a blend of grains naturally contaminated with fusarium mycotoxins on swine performance, brain regional neurochemistry, and serum chemistry and the efficacy of a polymeric glucomannan mycotoxin adsorbent. *J. Anim. Sci.* **2002**, *80*, 3257–3267.
13. Döll, S.; Dänicke, S.; Ueberschär, K.-H.; Valenta, H.; Schnurrbusch, U.; Ganter, M.; Klobasa, F.; Flachowsky, G. Effects of graded levels of *Fusarium* toxin contaminated maize in diets for female weaned piglets. *Arch. Anim. Nutr.* **2003**, *57*, 311–334.
14. Dänicke, S.; Valenta, H.; Klobasa, F.; Döll, S.; Ganter, M.; Flachowsky, G. Effects of graded levels of *Fusarium* toxin contaminated wheat in diets for fattening pigs on growth performance, nutrient digestibility, deoxynivalenol balance and clinical serum characteristics. *Arch. Anim. Nutr.* **2004**, *58*, 1–17.
15. Dänicke, S.; Valenta, H.; Gareis, M.; Lucht, H.W.; von Reichenbach, H. On the effects of a hydrothermal treatment of deoxynivalenol (DON)-contaminated wheat in the presence of sodium metabisulphite ($Na_2S_2O_5$) on DON reduction and on piglet performance. *Anim. Feed. Sci. Technol.* **2005**, *118*, 93–108.
16. Goyarts, T.; Dänicke, S.; Rothkötter, H.J.; Spilke, J.; Tiemann, U.; Schollenberger, M. On the effects of a chronic deoxynivalenol intoxication on performance, haematological and serum parameters of pigs when diets are offered either for *ad libitum* consumption or fed restrictively. *J. Vet. Med.* **2005**, *52*, 305–314.
17. Accensi, F.; Pinton, P.; Callu, P.; Abella-Bourges, N.; Guelfi, J.-F.; Grosjean, F.; Oswald, I. P. Ingestion of low doses of deoxynivalenol does not affect hematological, biochemical, or immune responses of piglets. *J. Anim. Sci.* **2006**, *84*, 1935–1942.

18. Cheng, Y.-H.; Weng, C.-F.; Chen, B.-J.; Chang, M.-H. Toxicity of different *Fusarium* mycotoxins on growth performance, immune responses and efficacy of a mycotoxin degrading enzyme in pigs. *Anim. Res.* **2006**, *55*, 579–590.
19. Gutzwiller, A.; Czegledi, L.; Stoll, P.; Bruckner, L. Effects of *Fusarium* toxins on growth, humoral immune response and internal organs in weaner pigs, and the efficacy of apple pomace as an antidote. *J. Anim. Physiol. Anim. Nutr.* **2007**, *91*, 432–438.
20. Waché, Y.J.; Valat, C.; Postollec, G.; Bougeard, S.; Burel, C.; Oswald, I.P.; Fravalo, P. Impact of deoxynivalenol on the intestinal microflora of pigs. *Int. J. Mol. Sci.* **2009**, *10*, 1–17.
21. Xiao, H.; Wu, M.M.; Tan, B.E.; Yin, Y.L.; Li, T.J.; Xiao, D.F.; Li, L. Effects of composite antimicrobial peptides in weanling piglets challenged with deoxynivalenol: I. Growth performance, immune function, and antioxidation capacity. *J. Anim. Sci.* **2013**, *91*, 4772–4780.
22. Shin, S.Y.; Kong, C.; Kim, I.H.; Kim, B.G. Effects of naturally produced dietary *Fusarium* mycotoxins on weaning pigs. *Am. J. Anim. Vet. Sci.* **2014**, *9*, 105–109.
23. Patience, J.F.; Myers, A.J.; Ensley, S.; Jacobs, B.M.; Madson, D. Evaluation of two mycotoxin mitigation strategies in grow-finish swine diets containing corn dried distillers grains with solubles naturally contaminated with deoxynivalenol. *J. Anim. Sci.* **2014**, *92*, 620–626.
24. Weaver, A.C.; See, M.T.; Kim, S.W. Protective effect of two yeast based feed additives on pigs chronically exposed to deoxynivalenol and zearalenone. *Toxins* **2014**, *6*, 3336–3353.
25. van Le Thanh, B.; Lessard, M.; Chorfi, Y.; Guay, F. The efficacy of anti-mycotoxin feed additives in preventing the adverse effects of wheat naturally contaminated with *Fusarium* mycotoxins on performance, intestinal barrier function and nutrient digestibility and retention in weanling pigs. *Can. J. Anim. Sci.* **2015**, *95*, 197–209.
26. Kong, C.; Shin, S.Y.; Park, C.S.; Kim, B.G. Effects of feeding barley naturally contaminated with *Fusarium* mycotoxins on growth performance, nutrient digestibility, and blood chemistry of gilts and growth recoveries by feeding a non-contaminated diet. *Asian-Australas. J. Anim. Sci.* **2015**, *28*, 662–670.
27. Alizadeh, A.; Braber, S.; Akbari, P.; Garssen, J.; Fink-Gremmels, J. Deoxynivalenol impairs weight gain and affects markers of gut health after low-dose, short-term exposure of growing pigs. *Toxins* **2015**, *7*, 2071–2095.
28. Wu, L.; Liao, P.; He, L.; Ren, W.; Yin, J.; Duan, J.; Li, T. Growth performance, serum biochemical profile, jejunal morphology, and the expression of nutrients transporter genes in deoxynivalenol (DON)-challenged growing pigs. *BMC Vet. Res.* **2015**, *11*, 144.
29. Kong, C.; Park, C.S.; Kim, B.G. Evaluation of a mycotoxin adsorbent in swine diets containing barley naturally contaminated with *Fusarium* mycotoxins. *Rev. Colomb. Cienc. Pec.* **2016**, *29*, 169–177.
30. van Le Thanh, B.; Lemay, M.; Bastien, A.; Lapointe, J.; Lessard, M.; Chorfi, Y.; Guay, F. The potential effects of antioxidant feed additives in mitigating the adverse effects of corn naturally contaminated with *Fusarium* mycotoxins on antioxidant systems in the intestinal mucosa, plasma, and liver in weaned pigs. *Mycotoxin Res.* **2016**, *32*, 99–116.
31. Jin, L.; Wang, W.; Degroote, J.; van Noten, N.; Yan, H.; Majdeddin, M.; van Poucke, M.; Peelman, L.; Goderis, A.; van De Mierop, K. Mycotoxin binder improves growth rate in piglets associated with reduction of toll-like receptor-4 and increase of tight junction protein gene expression in gut mucosa. *J. Anim. Sci. Biotechnol.* **2017**, *8*, 80.
32. Frobose, H.L.; Stephenson, E.W.; Tokach, M.D.; DeRouchey, J.M.; Woodworth, J.C.; Dritz, S.S.; Goodband, R.D. Effects of potential detoxifying agents on growth performance and deoxynivalenol (DON) urinary balance characteristics of nursery pigs fed DON-contaminated wheat. *J. Anim. Sci.* **2017**, *95*, 327–337.
33. Li, F.C.; Wang, J.Q.; Huang, L.B.; Chen, H.J.; Wang, C.Y. Effects of adding *Clostridium* sp. WJ06 on intestinal morphology and microbial diversity of growing pigs fed with natural deoxynivalenol contaminated wheat. *Toxins* **2017**, *9*, 383.
34. Li, R.; Li, Y.; Su, Y.; Shen, D.; Dai, P.; Li, C. Short-term ingestion of deoxynivalenol in naturally contaminated feed alters piglet performance and gut hormone secretion. *Anim. Sci. J.* **2018**, *89*, 1134–1143.
35. Reddy, K.E.; Song, J.; Lee, H.-J.; Kim, M.; Kim, D.-W.; Jung, H.J.; Kim, B.; Lee, Y.; Yu, D.; Kim, D.-W; et al. Effects of high levels of deoxynivalenol and zearalenone on growth performance, and hematological and immunological parameters in pigs. *Toxins* **2018**, *10*, 114.
36. Ebarb, S.M.; Fowler, C.M.; Xue, P.; Williams, S.B.; Peters, J.C.; Giesting, D.W. The impact of a sulfur-containing preservative blend on growth performance of growing pigs (30-100 kg) fed diets containing deoxynivalenol (DON). *J. Anim. Sci.* **2018**, *96* (Suppl. 2), 124 (Abstr.).
37. Sayyari, A.; Fæste, C.K.; Hansen, U.; Uhlig, S.; Framstad, T.; Schatzmayr, D.; Sivertsen, T. Effects and biotransformation of the mycotoxin deoxynivalenol in growing pigs fed with naturally contaminated pelleted grains with and without the addition of *Coriobacteriaceum* DSM 11798. *Food Addit. Contam.* **2018**, *35*, 1394–1409.

38. Li, X.; Guo, Y.; Zhao, L.; Fan, Y.; Ji, C.; Zhang, J.; Ma, Q. Protective effects of *Devosia* sp. ANSB714 on growth performance, immunity function, antioxidant capacity and tissue residues in growing-finishing pigs fed with deoxynivalenol contaminated diets. *Food Chem. Toxicol.* **2018**, *121*, 246–251.
39. Levesque, C.; Samuel, R.; Woyengo, T.; Herrick, K.; Thaler, R. Mitigating effects of DON-contaminated DDGS on pig performance. *J. Anim. Sci.* **2018**, *96* (Suppl. 3), 330–331 (Abstr.).
40. Acosta, J.A.; Jones, G.M.; Patience, J.F. 165 Impact of mycotoxin contamination of feed on growth performance and blood metabolites of growing pigs. *J. Anim. Sci.* **2019**, *97* (Suppl. 2), 93 (Abstr.).
41. Rho, Y.; Voth, C.; Buis, Q.; Trott, D.; Huber, L.-A.; Kiarie, E. 357 Evaluation of growth performance and physiology of nursery pigs fed deoxynivalenol (DON) contaminated feed supplemented with commercial feed additives (CFA). *J. Anim. Sci.* **2019**, *97* (Suppl. 2), 143–144 (Abstr.).
42. Liao, P.; Li, Y.; Li, M.; Chen, X.; Yuan, D.; Tang, M.; Xu, K. Baicalin alleviates deoxynivalenol-induced intestinal inflammation and oxidative stress damage by inhibiting NF-κB and increasing mTOR signaling pathways in piglets. *Food Chem. Toxicol.* **2020**, *140*, 111326.

Table S1. Experimental conditions in 42 studies.

Reference	DON source	DON, mg/kg	Initial BW, kg	Exp. period, d	ΔWG changes, %	ΔFI changes, %
Friend et al., 1983	Naturally contaminated wheat	2.00	123.0	51	−5.1	−3.8
		4.00	119.0	51	−21.4	−18.2
Young et al., 1983	Naturally contaminated corn	9.00	8.2	11	−72.2	−54.0
		1.34	7.0	21	−23.5	−22.6
		2.55	6.8	21	−44.1	−39.6
		5.12	6.9	21	−64.7	−62.3
		6.39	6.8	21	−67.6	−62.3
		7.83	7.1	21	−76.5	−67.9
		8.63	7.8	21	−61.8	−49.1
Chavez, 1984	Naturally contaminated wheat	1.73	6.9	56	−2.1	−2.4
		2.51	6.9	56	−18.1	−17.9
Cote et al., 1985	Naturally contaminated corn	3.10	10.0	35	−20.2	-
		5.80	9.6	35	−17.5	-
Lun et al., 1985	Naturally contaminated corn	10.50	8.3	21	−50.0	−44.4
Pollmann et al., 1985	Naturally contaminated wheat	0.90	7.7	21	2.9	6.3
		2.00	7.7	21	−17.1	−23.8
		2.80	7.7	21	−48.6	−34.9
		1.40	8.3	14	−2.9	−12.9
		0.90	60.5	42	−10.3	−14.4
		2.20	60.5	42	−18.4	−29.2
Foster et al., 1986	Naturally contaminated corn	4.70	27.5	49	−20.1	−17.9
		4.90	27.5	49	−29.8	−34.2
		4.80	27.5	49	−34.4	−34.5
		5.10	27.5	49	−36.3	−39.9
		5.10	27.5	49	−25.6	−23.2
		5.20	27.5	49	−37.5	−40.1
		4.70	27.5	49	−20.7	−16.4
		4.20	27.5	49	−20.3	−20.1
		4.70	27.5	49	−33.3	−36.7
		5.20	27.5	49	−29.8	−26.1
		3.70	27.5	49	−17.8	−23.0
		4.60	27.5	49	−26.2	−23.7
		3.30	27.5	49	−16.6	−13.0
		3.60	27.5	49	−20.3	−14.9
		2.10	27.5	49	−11.9	−10.5
		2.80	27.5	49	−25.2	−23.1
		3.80	27.5	49	−22.8	−22.6
Bergsjø et al., 1993	Naturally contaminated oats	0.70	20.9	94	−0.6	−0.5
		1.70	21.5	96	−3.7	−7.9
		3.50	22.0	95	−17.6	−15.0
He et al., 1993	Naturally contaminated corn	4.80	11.6	5	−56.7	−34.0

Reference	DON source	DON, mg/kg	Initial BW, kg	Exp. period, d	ΔWG changes, %	ΔFI changes, %
Rotter et al., 1995	Naturally contaminated corn	4.00	17.9	42	−13.0	−20.0
Smith et al., 1997	Naturally contaminated barley and wheat	4.40	8.2	21	−34.9	−29.6
		6.00	8.2	21	−39.8	−34.9
		7.50	8.2	21	−49.4	−43.3
		0.50	8.3	21	−2.2	1.3
		1.10	8.3	21	−16.7	−9.7
		1.90	8.3	21	−7.8	−8.9
		2.20	8.2	21	−3.3	−1.3
		2.40	8.2	21	−4.4	−2.8
		2.50	8.2	21	−12.2	−8.1
Swamy et al., 2002	Naturally contaminated corn and wheat	4.60	10.0	21	−34.6	−32.6
Döll et al., 2003	Naturally contaminated corn	0.80	12.4	36	3.6	1.3
		1.00	12.4	36	3.6	0.2
		1.90	12.4	36	−3.6	−5.1
		3.90	12.4	36	−19.2	−22.5
Dänicke et al., 2004	Naturally contaminated wheat	2.64	28.0	14	−20.1	−13.8
		4.41	28.0	14	−54.5	−40.4
		1.40	55.2	42	−4.1	−5.9
		2.70	54.6	42	−1.5	−4.3
Dänicke et al., 2005	Naturally contaminated wheat	3.80	12.3	21	−15.7	−15.3
Goyarts et al., 2005	Naturally contaminated wheat	6.15	26.0	77	−13.7	−14.8
Accensi et al., 2006	Naturally contaminated wheat	0.28	11.2	28	−5.7	0.3
		0.56	11.2	28	−2.9	−5.8
		0.84	11.2	28	3.9	1.5
Cheng et al., 2006	Naturally contaminated corn	1.00	8.9	36	−16.8	−28.1
Gutzwiller et al., 2007	Naturally contaminated wheat	3.10	10.0	35	−9.4	−10.1
Waché et al., 2009	Naturally contaminated wheat	2.70	29.6	28	−16.5	−9.9
Xioa et al., 2013	Purified DON	4.00	12.4	30	−27.1	−13.0
Shin et al., 2013	Naturally contaminated corn germ meal	0.53	5.0	28	−3.6	−2.7
		1.03	5.1	28	−6.7	−3.4
		1.53	5.0	28	−6.4	−3.9
Patience et al., 2014	Naturally contaminated DDGS	4.80	22.8	115	−12.0	−8.7
Weaver et al., 2014	Naturally contaminated corn	4.82	9.0	42	−33.5	−0.4
van Le Thanh et al., 2015	Naturally contaminated wheat	4.61	6.9	14	−41.0	−21.5

Reference	DON source	DON, mg/kg	Initial BW, kg	Exp. period, d	ΔWG changes, %	ΔFI changes, %
Kong et al., 2015	Naturally contaminated barley	6.10	33.0	14	−3.3	−4.6
		7.70	33.0	14	−41.1	−21.4
		14.60	34.8	14	−80.4	−38.1
Wu et al., 2015	Naturally contaminated corn	3.00	16.3	21	−0.9	2.2
		6.00	16.3	21	−10.2	−14.8
		12.00	16.3	21	−43.9	−41.6
Alizadeh et al., 2015	Purified DON	0.90	7.9	10	−31.0	−3.2
Kong et al., 2016	Naturally contaminated barley	2.94	62.1	14	−30.7	−15.6
van Le Thanh et al., 2016	Naturally contaminated corn	3.13	7.1	17	−10.5	−8.3
Li et al., 2017	Naturally contaminated wheat	1.90	56.4	35	−37.4	−22.4
Frobose et al., 2017	Naturally contaminated wheat	4.10	13.4	21	−18.9	−12.0
Jin et al., 2017	Purified DON	3.00	7.3	37	−2.0	−3.0
Reddy et al., 2018	Purified DON	8.00	19.6	28	−30.2	−7.1
Sayyari et al., 2018	Naturally contaminated oats	0.92	10.9	35	4.5	6.6
		2.20	10.3	35	−3.9	−5.9
		5.00	11.9	35	−5.2	−3.4
Li et al., 2018	Naturally contaminated rice	2.85	48.1	18	−18.7	−12.6
Li et al., 2018	Naturally contaminated barley	1.20	8.9	21	−4.1	−4.4
		2.13	9.0	21	−10.7	−17.4
		3.17	9.0	21	−13.3	−25.3
Ebarb et al., 2018	Naturally contaminated DDGS	4.00	29.5	70	−14.3	−14.7
Levesque et al., 2018	Naturally contaminated DDGS	0.90	21.5	21	−9.8	-
Rho et al., 2019	Naturally contaminated corn	2.30	10.5	28	−1.3	0.3
Acosta et al., 2019	Naturally contaminated corn	1.80	73.1	28	−51.7	−48.1
Liao et al., 2020	Naturally contaminated corn	4.00	6.5	14	−15.0	−11.3

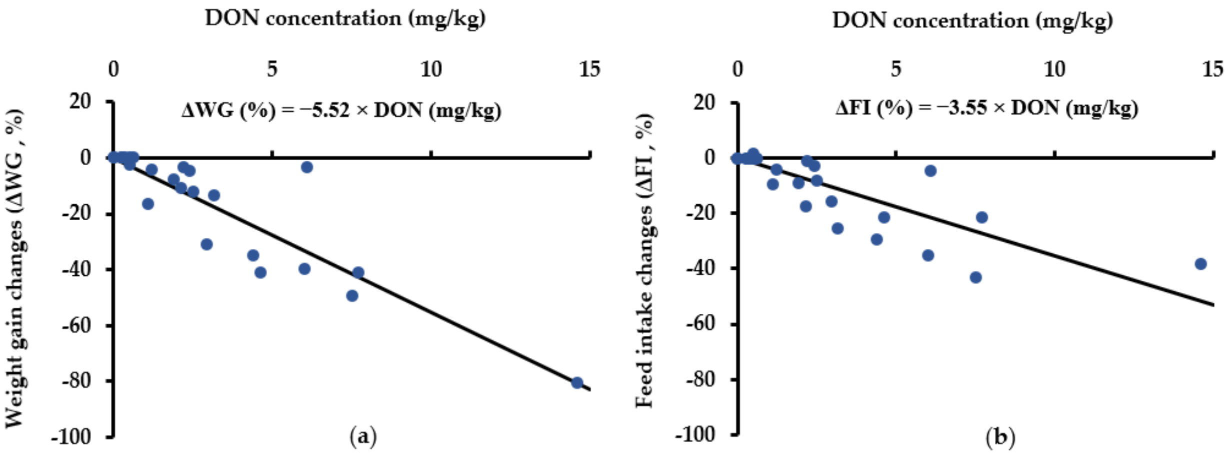

Figure S1. Regression equations for predicting weight gain changes (ΔWG, %) and feed intake changes (ΔFI, %) of pigs by dietary deoxynivalenol based on ELISA analysis method (n = 24). The y-intercept of new equations was forced to zero. (**a**) ΔWG = −5.52 × DON with SE = 0.41, $r^2 = 0.88$, and $p < 0.001$. (**b**) ΔFI = −3.55 × DON with SE = 0.38, $r^2 = 0.78$, and $p < 0.001$. Dietary DON concentrations ranged from 0 to 14.6 mg/kg.

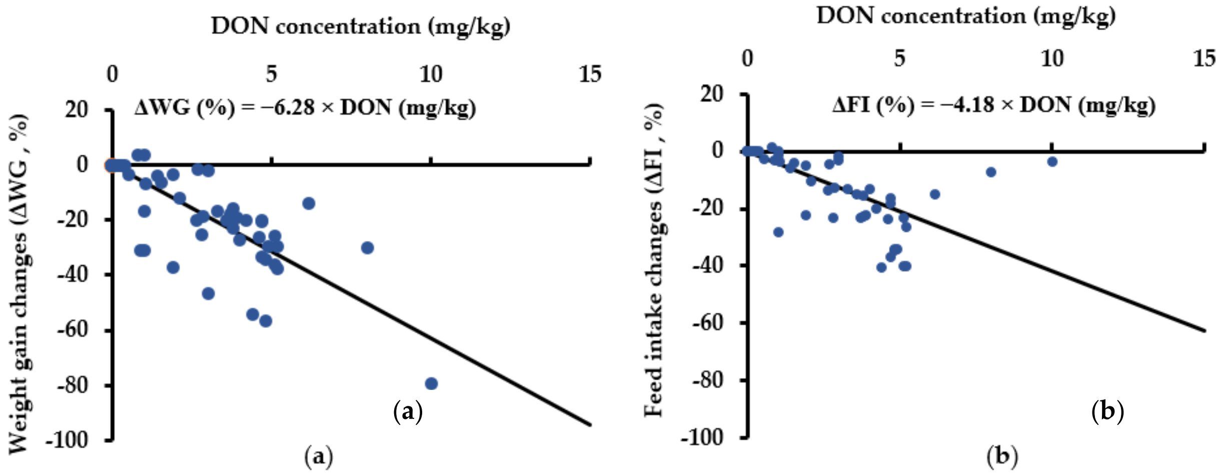

Figure S2. Regression equations for predicting weight gain changes (ΔWG, %) and feed intake changes (ΔFI, %) of pigs by dietary deoxynivalenol based on HPLC analysis method (n = 57). The y-intercept of new equations was forced to zero. (**a**) ΔWG = −6.28 × DON with SE = 0.43, $r^2 = 0.79$, and $p < 0.001$. (**b**) ΔFI = −4.18 × DON with SE = 0.39, $r^2 = 0.67$, and $p < 0.001$. Dietary DON concentrations ranged from 0 to 10 mg/kg.

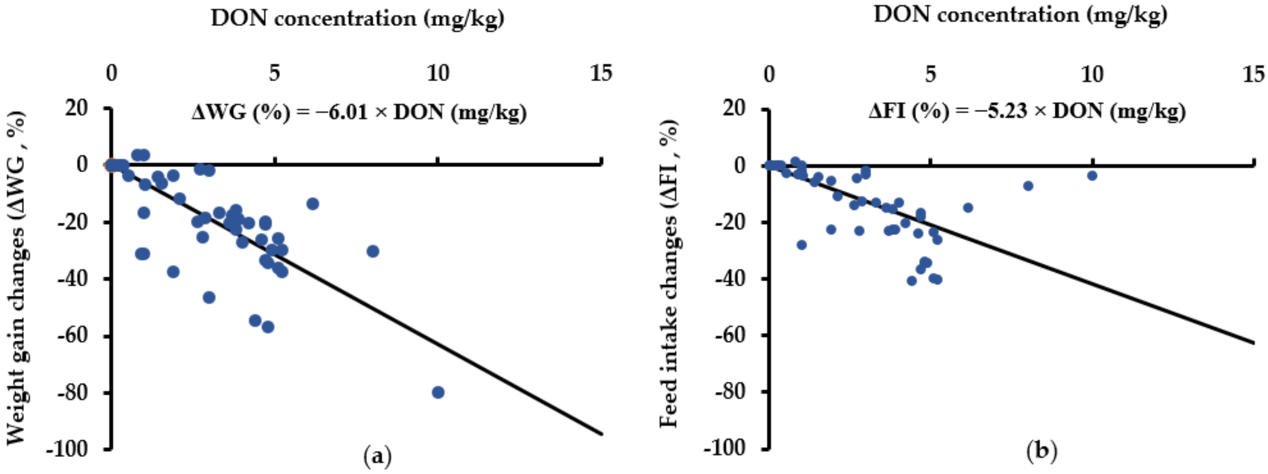

Figure S3. Regression equations for predicting weight gain changes (ΔWG, %) and feed intake changes (ΔFI, %) of pigs by dietary deoxynivalenol based on LC-MS/MS analysis method (n = 62). The y-intercept of new equations was forced to zero. (**a**) ΔWG = −6.01 × DON with SE = 0.45, $r^2 = 0.75$, and $p < 0.001$. (**b**) ΔFI = −5.23 × DON with SE = 0.42, $r^2 = 0.73$, and $p < 0.001$. Dietary DON concentrations ranged from 0 to 12 mg/kg.

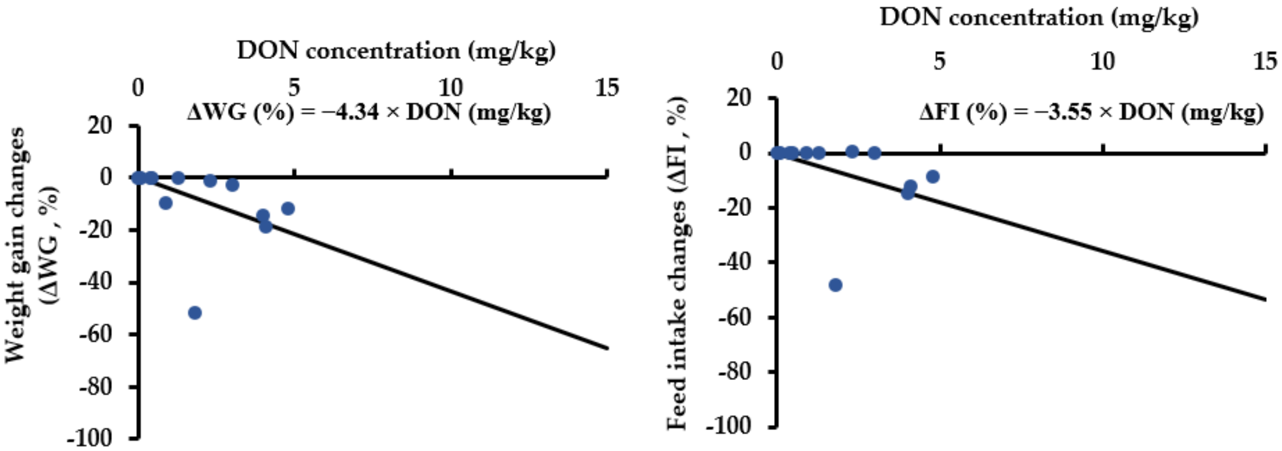

Figure S4. Regression equations for predicting weight gain changes (ΔWG, %) and feed intake changes (ΔFI, %) of pigs by dietary deoxynivalenol based on unknown analysis method (n = 13). The y-intercept of new equations was forced to zero. (**a**) ΔWG = −4.34 × DON with SE = 1.89, $r^2 = 0.37$, and $p = 0.047$. (**b**) ΔFI = −3.55 × DON with SE = 1.79, $r^2 = 0.30$, and $p = 0.079$. Dietary DON concentrations ranged from 0 to 4.8 mg/kg.
